# Targeting the Dynamics Between TAMs and CAR‐T Cells in Solid Tumor Therapies

**DOI:** 10.1002/advs.202515700

**Published:** 2025-11-21

**Authors:** Xiang Zhang, Hao Zheng, Zi‐Chang Liu, Ling‐Jie Luo, Xiao Dong, Xin‐Gang Cui, Yan‐Wei Wu, Liang Chen

**Affiliations:** ^1^ School of Medicine Shanghai University Shanghai 200444 China; ^2^ Department of Urology, Xinhua Hospital Shanghai Jiao Tong University School of Medicine Shanghai 200092 China; ^3^ Shanghai Tenth People's Hospital Shanghai 200072 China; ^4^ Institute of Artificial Intelligence and Biomanufacturing Shanghai University Shanghai 200444 China

**Keywords:** CAR‐T cells, solid tumors, tumor microenvironment, tumor‐associated macrophages

## Abstract

Although CAR T‐cell therapy has transformed treatment outcomes for patients with haematological malignancies, the existing barriers in solid tumors treatment remain due to TAM immunosuppression. This review explores the work of others on the interplay between M2‐like immunosuppressive TAMs and CAR‐T cells in the tumor microenvironment. The ways TAMs impair the effector functions of CAR‐T cells are described mediated by secretion of cytokines, immune checkpoints, metabolites, and detrimental post‐translational modification. New concept therapeutic approaches are designed to these interactions for improving the efficacy of CAR‐T cells in solid tumors. With an emphasis on novel strategies to counteract TAM immunosuppression, this review aims to reshape the perspective on the utility and effectiveness of CAR‐T therapy in solid tumors and, consequently, extend the reach of a highly promising therapeutic approach.

## Graphical abstract

This review highlights the critical role of tumor‐associated macrophages (TAMs) in affecting CAR‐T cell therapy effectiveness in solid tumors. TAMs contribute to immune suppression through cytokine secretion, immune checkpoint activation, and metabolic reprogramming, leading to CAR‐T cell exhaustion and reduced efficacy. The article explores strategies to counteract TAM‐driven immune evasion and improve CAR‐T cell outcomes in solid tumors.

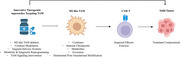

## 1 Introduction

### 1.1 Overview of CAR‐T Therapy in Cancer

Cancer immunotherapy utilizes the body's defense mechanisms to fight cancer, with Chimeric Antigen Receptor (CAR) T‐cell therapy being a revolutionary treatment. By modifying T‐cells to target and destroy tumor cells,^[^ ^1^ ^]^ CAR‐T therapy has shown remarkable success in blood cancers^[^ ^2^ ^]^ and is now being explored for solid tumors.^[^ ^3^ ^]^ In hematological malignancies, second‐generation CAR‐T cells like CD19‐targeting CAR‐T cells have been highly effective in treating acute lymphoblastic leukemia (ALL) and diffuse large B‐cell lymphoma (DLBCL)^.[^ ^4^ ^]^ These therapies enable T‐cells to target specific tumor antigens, leading to powerful immune responses, with CD19‐targeting CAR‐T cells achieving profound remission in relapsed ALL.^[^ ^5^ ^]^ Additionally, BCMA‐targeting CAR‐T cells have been approved for multiple myeloma, offering new hope for patients.^[^ ^6^ ^]^ The success in these settings is due to CAR‐T cells' ability to circulate and persist in the bloodstream, a more favorable environment than the complex tumor microenvironment in solid tumors.

Unlike blood cancers, solid tumors have a highly organized yet antagonistic tumor microenvironment (TME), which impairs the efficacy of CAR‐T therapies.^[^ ^7^ ^]^ Addressing the unique challenges in solid tumors is key to extending the success of CAR‐T therapies beyond hematological cancers.

### 1.2 Tumor‐Associated Macrophages (TAMs) in Solid Tumors

CAR‐T cell therapy faces significant challenges in solid tumors due to the complex TME, which includes a dense extracellular matrix (ECM), low oxygen levels, acidic conditions and antigen heterogeneity that hinder CAR‐T cell infiltration and function.^[^ ^8^, ^9^, ^10^ ^]^

The TME is also rich in immune suppressive cells such as regulatory T cells (Tregs), myeloid‐derived suppressor cells (MDSCs), and most notably, TAMs.^[^ ^11^ ^]^ Macrophages in the TME are classified as M1 (pro‐inflammatory, anti‐tumor) and M2 (immunosuppressive, pro‐tumor). M1 macrophages, activated by IFN‐γ or bacterial products, release cytokines like TNF‐α, IL‐1β, and IL‐12 to activate effector T cells and promote tumor immunity, though IFN‐γ also induces PD‐L1, limiting immune efficacy.^[^ ^12^ ^]^ In contrast, M2‐like macrophages dominate the TME, secreting growth factors TGF‐β and immunosuppressive cytokines like IL‐10 that inhibit cytotoxic T cells and promote regulatory T cell activity, aiding tumor progression.^[^ ^13^ ^]^

Actually, the traditional M1/M2 classification of macrophages is becoming outdated, with recent research shifting focus to macrophage ontogeny and activation. This approach highlights their dynamic nature and role in tumor progression. Tissue‐resident macrophages, marked by CD206, can shift phenotypically in response to local cues, complicating tumor dynamics.^[^ ^14^ ^]^ This has led to therapies targeting macrophage activation, including metabolic reprogramming, to overcome their immunosuppressive effects and boost anti‐tumor immunity.^[^ ^15^, ^16^ ^]^

In addition to TAMs, myeloid‐derived suppressor cells (MDSCs) are critical immunosuppressive populations in the solid tumor TME, which share many features with TAMs. TAMs, characterized by CD68, CD163, CD206, and TGF‐β, are critical for tumor progression.^[^ ^17^ ^]^ MDSCs, which are immature myeloid cells that fail to fully differentiate, are categorized into monocytic MDSCs (M‐MDSCs), marked by CD11b^+^/Gr‐1^int^/Ly6C^high^/Ly6G^−^, and granulocytic MDSCs (G‐MDSCs), marked by CD11b^+^/Gr‐1^high^/Ly6C^−^/Ly6G^high^. Both MDSC subsets secrete IL‐10, TGF‐β, and reactive oxygen species (ROS), contributing to immune suppression.^[^ ^18^ ^]^

### 1.3 Evolution of CAR‐T Cells

First‐generation CAR‐T cells, designed with a TCR signaling domain and CD3ζ chain, were effective in hematologic malignancies but were simpler compared to modern systems. Second‐generation CAR‐T cells introduced co‐stimulatory domains like CD28 or 4‐1BB, improving persistence and efficacy. Third‐generation CAR‐Ts added multiple co‐stimulatory signals to enhance functionality.^[^ ^19^ ^]^ Dual‐targeted CAR‐T cells, which target both tumor antigens and macrophages, aim to improve therapeutic outcomes, as seen in hepatocellular carcinoma.^[^ ^20^ ^]^

The 2.0 era introduces advanced models like SUPRA (Split, Universal, and Programmable RNA‐Assisted) CARs and in vivo engineered CAR‐T cells, further refining CAR‐T therapy by enhancing specificity, minimizing off‐target effects and reducing treatment costs. SUPRA CARs feature an inducible co‐stimulatory system for better solid tumor targeting,^[^ ^21^ ^]^ while in vivo approaches allow real‐time adjustments.^[^ ^22^ ^]^ These innovations highlight the importance of understanding CAR‐T interaction with TAMs to optimize therapy for solid tumors.

## 2 TAM‐CAR‐T Interactions Mechanisms

TAMs play a key role in creating an immunosuppressive TME that hinders CAR‐T cell therapy. Because CAR‐T cells are still T cells, therefore they undergo similar mechanistic regulation as “regular” T cells, including epigenetic and metabolic regulation, exhaustion, impact of cytokine and chemokine networks. Of note, CAR‐T cells face unique exhaustion mechanisms not seen in regular T cells due to their engineered persistence and prolonged antigen stimulation. This continuous activation leads to metabolic shifts, reduced cytokine production, impaired cytotoxicity, and increased inhibitory receptors like PD‐1, CTLA‐4, and TIM‐3.^[^ ^23^ ^]^ Epigenetic changes, such as histone modifications, further contribute to CAR‐T dysfunction by dysregulating genes essential for activation, survival, and memory.^[^ ^24^ ^]^ Unlike regular T cells, which are activated by external stimuli, CAR‐T cells experience sustained activation within the immunosuppressive tumor microenvironment, intensifying epigenetic alterations and exhaustion.^[^ ^25^ ^]^

Additionally, TAMs affect the TME through various mechanisms such as cytokine secretion, immune checkpoint expression, metabolic reprogramming, and exosome release. TAM‐induced post‐translational modifications (PTMs)—including phosphorylation, ubiquitination, acetylation, and glycosylation—disrupt CAR‐T cell signaling, leading to exhaustion and reduced antitumor activity (**Table** **1**).

**Table 1:** Mechanisms of CAR‐T and TAM Interactions.

Mechanism	Description	Impact on CAR‐T Therapy
**Cytokine Secretion (IL‐10, TGF‐β)**	TAMs secrete cytokines suppress CAR‐T cell expansion and cytotoxic activity by downregulating inflammatory cytokines.	Reduces CAR‐T cell efficacy by inhibiting the immune response and promoting an immunosuppressive environment.
**Immune Checkpoints (PD‐L1, CTLA‐4)**	TAMs express immune checkpoint molecules which bind to PD‐1 on CAR‐T cells, inducing exhaustion and suppressing their activity.	Promotes CAR‐T cell exhaustion and dysfunction, hindering their anti‐tumor potential.
**Metabolic Reprogramming**	TAMs promote a glycolytic metabolism, increasing lactate levels, which impairs CAR‐T cell energy production and function.	Limits CAR‐T cell activation, survival, and cytotoxicity by creating a competitive, nutrient‐deprived environment.
**Exosome Secretion**	TAM‐derived exosomes carry immune checkpoint molecules and cytokines that modulate CAR‐T cell behavior, contributing to immune evasion and therapy resistance.	Contributes to CAR‐T cell exhaustion and resistance by altering signaling pathways.
**TAM and MDSC Interaction**	TAMs collaborate with MDSCs to recruit more immunosuppressive cells into the tumor, worsening the TME.	Amplifies the immunosuppressive effects, decreasing CAR‐T cell persistence and effectiveness.
**TAM‐Derived Adenosine**	TAMs convert ATP into adenosine, which binds to the A2A receptor on CAR‐T cells, leading to increased cAMP levels and suppressed TCR signaling.	Inhibits CAR‐T cell function by reducing cytokine production and increasing the suppressive environment.
**Hypoxia and Metabolic Competition**	TAMs contribute to the hypoxic conditions in the TME, exacerbating CAR‐T cell metabolic stress.	Impairs CAR‐T cell proliferation and function by promoting glycolysis and lactate accumulation.
**PTMs**	TAMs induce various PTMs (phosphorylation, ubiquitination, acetylation, glycosylation) that alter CAR‐T cell signaling pathways, leading to dysfunction and exhaustion.	Modifies CAR‐T cell function by disrupting key signaling pathways, impairing survival, activation, and cytotoxic activity.

### 2.1 Signal Pathways and Immune Modulation

TAMs and CAR‐T cell interactions involve complex signaling pathways, including cytokine secretion, checkpoint molecule expression, and metabolic changes. These factors create an immunosuppressive environment that undermines the effectiveness of CAR‐T therapies in solid tumors.

#### 2.1.1 Cytokine Secretion

TAMs, specifically those of the M2 variety, release several cytokines responsible for generating an immunosuppressive environment that diminishes the effectiveness of CAR‐T cell therapy. Cytokines inhibit CAR‐T cell expansion and cytotoxic activity directly by downregulating the expression of essentials inflammatory cytokines such as IFN‐γ^[^ ^26^ ^]^ and cytotoxic molecules including granzyme B and perforin.^[^ ^27^ ^]^ In addition, IL‐6 serves a variety of functions in immune system regulation. It also helps to maintain and increase the numbers of regulatory T cells (Tregs), which further inhibit the activity of CAR‐T cells within the tumor microenvironment.^[^ ^28^ ^]^ The presence of high levels of IL‐6 has a particularly strong association with the release of cytokines syndrome (CRS) which is an illness of great concern during CAR‐T therapy.^[^ ^29^ ^]^

**Figure 1:**
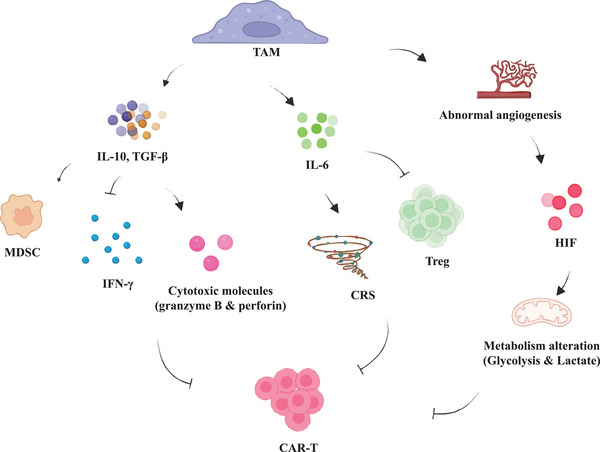
Immunomodulatory Effects of TAMs in the Tumor Microenvironment on CAR‐T Cell Function.

Beyond immune suppression, TAMs contribute to the development of CRS through the interaction of CD40 on TAMs with CD40L on CAR‐T cells. This interaction activates the release of pro‐inflammatory cytokines, which is a critical mechanism in driving CRS.^[^ ^30^ ^]^ Within the solid tumor microenvironment, TAMs collaborate with MDSCs, worsening the recruitment of additional immunosuppressive cells toward the tumor.^[^ ^31^ ^]^ This synergy amplifies the immunosuppressive environment by producing further inhibitory factors, thereby increasing the tolerogenic state and exacerbating the dysfunction of CAR‐T cells. The persistence of this inflammatory environment intensifies the severity of CRS, further diminishing the therapeutic efficacy of CAR‐T cells^[^ ^32^ ^]^ (**Figure** **1**).

To counteract these negative effects, CAR‐engineered natural killer (NK) cells have been proposed as a solution. CAR‐NK cells can help reduce CRS and enhance CAR‐T cell therapy outcomes by targeting and eliminating immunosuppressive cells like TAMs and MDSCs. This reduces inhibitory signals, alleviates the pro‐inflammatory cytokines released during the CD40‐CD40L interaction, and restores CAR‐T cell function by modulating the immune environment, improving the therapeutic response.^[^ ^33^ ^]^

#### 2.1.2 Metabolic Alterations

TAMs also impact the metabolic condition of CAR‐T cells which is vital for their survival, proliferation, and cytotoxicity. TME of solid tumors usually has low oxygen tension (hypoxia) as well as changed concentration of nutrients, all of which can hinder the metabolism of CAR‐T cells^[^ ^34^ ^]^ (see Figure 1 again).

**Figure 2:**
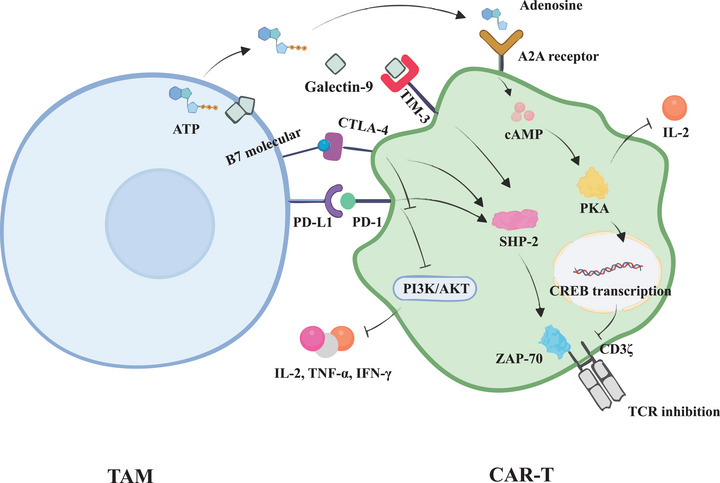
TAM‐mediated immunosuppression of CAR‐T cells through multiple inhibitory pathways.

For instance, adenosine released by TAM binds to A2A receptor on CAR‐T cells inducing an immunosuppressive T‐cell‐suppressive phenotype that decreases T‐cell activity.^[^ ^35^ ^]^ This cellular alteration causes diminished energy reserves in CAR‐T cells, diminished cytokine release, and weakened cytotoxic capacity.^[^ ^36^ ^]^ In addition, TAMs may secrete lactate and additional CAR‐T cell suppressor metabolic byproducts that inhibit activation and proliferation of CAR‐T cells in the TME^[^ ^37^, ^38^ ^]^ (**Figure** **2**).

This figure shows how TAMs interact with the TME to influence CAR‐T cell activity. TAMs produce IL‐10 and TGF‐β, leading to MDSC immigration and immune suppression characterized by an inadequate IFN‐γ response and cytotoxic molecule (granzyme B and perforin) activation. At the same time, TAM‐derived IL‐6 increases Treg expansion and suppressive activity. An immune system imbalance leads to metabolic dysregulation and hypoxia which exacerbates CRS. Due to the abnormal angiogenesis caused by TAMs, hypoxia is also induced which activates HIFs, driving metabolic changes in CAR‐T cells like increased glycolysis with higher lactate levels. These altered metabolism undermine the functionality of CAR‐T cells, making them less effective at tumor eradication.

#### 2.1.3 Checkpoint Molecule Expression

A primary way in which TAMs affect the activity of CAR‐T cells is by the expression of immune checkpoints. TAMs express PD‐L1, which interacts with the PD‐1 receptor present on CAR‐T cells, triggering inhibitory signals that reduce cytotoxic activity.^[^ ^39^ ^]^ This engagement limits the activation of CAR‐T cells and induces a state of T‐cell exhaustion, characterized by impaired proliferation and reduced production of effector cytokines. In response to this, third‐generation CAR‐T cells, which incorporate the CD28 co‐stimulatory domain, have been designed to counteract these inhibitory signals. The CD28 domain enhances CAR‐T cell activation by providing additional co‐stimulation, which overrides the PD‐1/PD‐L1‐mediated exhaustion and restores CAR‐T cell function, improving persistence and anti‐tumor activity.^[^ ^40^ ^]^

In addition to PD‐L1, TAMs may express other immune checkpoint molecules, including CTLA‐4 and TIM‐3. CTLA‐4 binds to B7 molecules on antigen‐presenting cells, competing with CD28 for co‐stimulation, and thus reducing CAR‐T cell activation and proliferation. However, the CD28 domain in third‐generation CAR‐T cells can compensate for this inhibition, maintaining robust activation. Furthermore, TIM‐3 is another inhibitory receptor that suppresses CAR‐T cell function by promoting apoptosis and lowering anti‐tumor responses (Figure 2). Third‐generation CAR‐T cells, through CD28 co‐stimulation, are more resilient to these pathways, thereby enhancing their therapeutic potential in solid tumors.^[^ ^41^ ^]^ Future research into these co‐inhibitory mechanisms and the development of strategies to resist them, such as dual checkpoint inhibitors or engineering CAR‐T cells, could provide promising approaches to overcoming the limitations of CAR‐T therapy in solid tumors.

TAMs primarily express PD‐L1, but they also express B7 molecules (CD80, CD86) and galectin‐9, which can bind to the CTLA‐4 and TIM‐3 receptors on CAR‐T cells, respectively.^[^ ^42^ ^]^ The binding of B7 molecules to CTLA‐4 competes with CD28 co‐stimulation, thereby reducing CAR‐T cell activation and proliferation.^[^ ^43^ ^]^ Similarly, galectin‐9 binding to TIM‐3 on CAR‐T cells can promote exhaustion by triggering inhibitory signals that impair CAR‐T cell function (Figure 2).^[^ ^44^ ^]^ Future research into these co‐inhibitory mechanisms and the development of strategies to resist them, such as dual checkpoint inhibitors or engineering CAR‐T cells, could provide promising approaches to overcoming the limitations of CAR‐T therapy in solid tumors.

TAM, via various mechanisms, suppresses CAR‐T cell functionality. TAMs produce PD‐L1 that binds to CAR‐T cells' PD‐1 receptor, activating SHP‐2. This activation deactivates ZAP‐70, which in turn TCR signaling inertia in T cells. Simultaneously, PD‐1 engagement to the receptor inhibits PI3K/AKT pathway signaling, further reducing effector T cell functions through diminished secretion of IL‐2, TNF‐α, and IFN‐γ indicative of T cell activity. Further, the TAMs mediates the conversion of ATP to adenosine which subsequently binds to A2A receptor on CAR‐T cells. This binding increases cAMP concentration in the cell, cascades that lead to protein kinase A (PKA) activation and subsequent CREB transcriptional activity. These alterations result in an additional suppression of TCR signaling and a reduction in IL‐2 production, worsening the already suppressive environment. TAMs also express B7 molecules (CD80, CD86) that interact with CTLA‐4 on T cells, which add another layer of inhibition that competes with CD28 co‐stimulatory signals, thus decreasing the CAR‐T cell activation and proliferation. Moreover, TAMs can bind galectin‐9 to TIM‐3 on CAR‐T cells, leading to the additional SHP‐2 driven TCR signaling inhibition.

#### 2.1.4 Role of TAM‐Derived Exosomes in CAR‐T Dysfunction

One largely overlooked aspect of TAM‐mediated CAR‐T cell dysfunction is the role of TAM‐derived exosomes in modulating CAR‐T cell behavior. Exosomes are small vesicles that carry bioactive molecules such as proteins, lipids, and RNAs, and are secreted by TAMs into the tumor microenvironment. Recent studies suggest that TAM‐derived exosomes could directly affect CAR‐T cell function, contributing to immune evasion and resistance to therapy. These exosomes carry proinflammatory cytokines, immune checkpoint molecules, and even specific microRNAs that regulate CAR‐T cell exhaustion. Targeting exosome biogenesis or blocking specific exosomal molecules could represent a novel approach to prevent TAMs from hijacking CAR‐T cell signaling. By incorporating exosome modulation into CAR‐T therapies, we may be able to restore CAR‐T cell activity and enhance their tumor‐killing potential in solid tumors. This approach is novel and may lead to the development of combination therapies involving exosome inhibitors and CAR‐T cells for better clinical outcomes in solid tumors.

#### 2.1.5 Bidirectional Crosstalk

TAMs and CAR‐T cells interact reciprocally, with some signaling pathways activated in CAR‐T cells that affect TAM function. For instance, CAR‐T cells can activate TAMs by IFN‐γ release, which subsequently augments pro‐inflammatory cytokine secretion from TAMs.^[^ ^45^ ^]^ Nonetheless, the presence of immunosuppressive factors often limits the duration of this inflammatory response, creating a feedback loop that ultimately suppresses the activity of the CAR‐T cells.^[^ ^46^ ^]^

### 2.2 Post‐Translational Modifications Affecting CAR‐T Functionality

TAMs have the potential to impact the activity of CAR‐T cells considerably by inducing PTMs of the proteins that govern the survival, proliferative expansion, and cytotoxic activity of the T cells.^[^ ^47^ ^]^ PTMs are crucial as regulatory mechanisms managing a range of cellular activities such as signal reception, immunological response, and cellular metabolism.^[^ ^48^ ^]^ Related to CAR‐T cells, TAM‐induced PTMs cause immune escape and T‐cell exhaustion, leading to diminished therapeutic effectiveness within TME.^[^ ^49^ ^]^

#### 2.2.1 Phosphorylation and Signal Transduction

**Figure 3:**
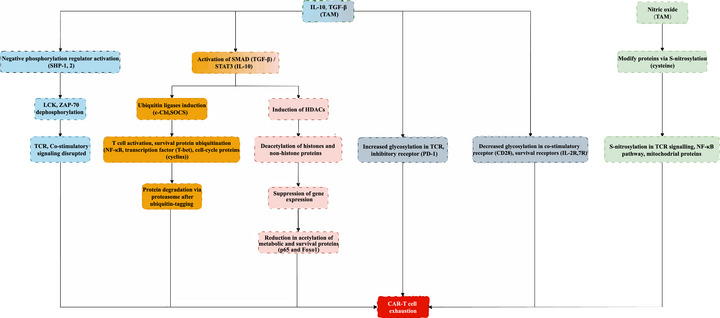
Mechanisms of CAR‐T Cell Exhaustion Induced by TAM‐Secreted Factors.

One of the most frequent and important PTMs that TAMs can induce in CAR‐T cells is phosphorylation, which is a modification that controls signal transduction pathways important for T‐cell activation and function.^[^ ^50^ ^]^ TAM‐secreted cytokines inhibit TCR signaling by triggering phosphorylation of key kinases like ZAP‐70 and LCK, disrupting T‐cell activation.^[^ ^51^, ^52^ ^]^ Nonetheless, the presence of TAM‐derived inhibitory signals alters this phosphorylation process. In particular, excessive phosphorylation of negative regulators like SHP‐1 and SHP‐2 severely impairs CAR‐T cell activation and effector functions^[^ ^53^ ^]^ (**Figure** **3**). Moreover, the phosphorylation of inhibitory receptors such as PD‐1, which is overexpressed on CAR‐T cells within the TME, further attenuates the CAR‐T cell response.^[^ ^54^ ^]^ The accumulation of these phosphorylation events leads to T‐cell exhaustion, marked by decreased proliferation and increased impairment of cytotoxicity, greatly diminishing the efficacy of CAR‐T cell therapy.^[^ ^55^ ^]^

#### 2.2.2 Ubiquitination and Proteasomal Degradation

Ubiquitination, which marks proteins for destruction by the proteasome, is another important PTM that regulates CAR‐T cell activity. Within the TME, TAMs can modulate the expression of certain ubiquitin ligases.^[^ ^56^ ^]^ A key target of TAM‐induced ubiquitination is the transcription factor NF‐κB. NF‐κB is responsible for anti‐inflammatory cytokine production and the survival of immune cells.^[^ ^57^, ^58^ ^]^ Signal exposure from TAM can influence the activity of ubiquitin ligases, leading to the degradation of proteins that maintain CAR‐T cell survival and activation.^[^ ^59^ ^]^ For example, the degradation of NF‐κB components may lead to a reduction in the production of inflammatory cytokines such as IFN‐γ, thereby hindering CAR‐T cells from mounting an effective antitumor response.^[^ ^60^ ^]^ Furthermore, the proteins connected to cell‐cycle regulation like cyclins may also undergo targeted degradation through the proteasome which may impair CAR‐T cell proliferation and persistence^[^ ^61^ ^]^ (Figure 3).

#### 2.2.3 Acetylation and Chromatin Remodeling

The addition of acetyl groups to the histones and non‐histone proteins is one more PTM that regulates the function of CAR‐T cells. In the presence of TAMs, cytokines serve to activate the histone deacetylases (HDACs) in CAR‐Ts. As a consequence, they bring about remodeling of the chromatin to a repressive state suppressing the active genes for T cell activation, differentiation, and cytokine production.^[^ ^62^ ^]^

Histone acetylation activates gene expression while deacetylation suppresses it, contributing to the transcriptional silencing that leads to CAR‐T cell exhaustion and dysfunction.^[^ ^63^ ^]^ Furthermore, TAMs have a role in regulating the acetylation of important metabolic and survival proteins in T‐cells, which include p65 of the NF‐κB pathway and Foxo1.^[^ ^64^, ^65^ ^]^ Reduced acetylation of these proteins diminishes CAR‐T cell viability and function, further amplifying immune evasion in TME^[^ ^66^ ^]^ (Figure 3).

#### 2.2.4 Glycosylation and Receptor Signaling

An additional PTM that may alter CAR‐T activity is glycosylation, which is defined as the addition of sugar moieties to a protein. TAMs are capable of altering the glycosylation of some of the major T cell receptors on CAR‐T cells, including the TCR and co‐stimulatory receptors like CD28.^[^ ^67^ ^]^ Different forms of glycosylation can change the binding affinity of these receptors and influence their signaling pathways. As an example, modified glycosylation on PD‐1 increases its interaction with PD‐L1 expressed on TAMs which strengthens the inhibitory signaling resulting in CAR‐T cell exhaustion.^[^ ^68^ ^]^

Moreover, TAM‐secreted factors can alter the glycosylation of cell survival receptors like IL‐2R and IL‐7R, modulating the responsiveness of CAR‐T cells to growth factors and altering their persistence within the TME. Changes in these glycosylation patterns can hinder the expansion and cytotoxicity of CAR‐T cells, severely impacting the efficacy of therapy^[^ ^69^ ^]^ (Figure 3).

#### 2.2.5 S‐nitrosylation and Immune Suppression

S‐nitrosylation, the addition of a nitric oxide (NO) group to cysteine residues of proteins, is a form of PTM that is usually impacted by TAM‐derived nitric oxide. This change leads to modification of the immune proteins in CAR‐T cells.^[^ ^70^ ^]^ For instance, S‐nitrosylation of TCR signaling and NF‐κB signaling proteins may blunt the responsiveness of CAR‐T cells to tumor cells.^[^ ^71^, ^72^ ^]^ TAM‐derived nitric oxide may also contribute to the S‐nitrosylation of proteins that participate in mitochondria‐ and metabolism‐related functions, hindering the energy production necessary for sustaining the CAR‐T cell's survival and cytotoxic activity^[^ ^73^, ^74^ ^]^ (Figure 3).

This flow chart depicts the intricate processes triggered by TAM‐secreted IL‐10 and TGF‐β culminating into CAR‐T cell exhaustion. Disruption of phosphorylation (depicted in blue) with SHP‐1 and SHP‐2 creates a blockade on the phospho‐ LCK and ZAP‐70, which subsequently halts the TCR and co‐stimulatory signaling cascade. At the same time, the SMAD (TGF‐β) and STAT3 (IL‐10) pathways are activated, stimulating the expression of ubiquitin ligases, particularly c‐Cbl and SOCS (indicated in yellow), which labeled NF‐κB, T‐bet, and cyclins for proteasomal degradation. This process occurs simultaneously with the induction of HDACs which deacetylate histones and non‐histone proteins (shown in pink), reducing gene expression and the acetylation of critical metabolic and survival effector proteins. In addition, patterns of glycosylation are altered (shown in grey), where the increased glycosylation of TCR and PD‐1 boost inhibitory signaling, while the loss of glycosylation in CD28, IL‐2R, and IL‐7R disrupts activation and survival signals. Also, TAM S‐nitrosylates (depicted in green) proteins which impacts TCR signaling along with the NF‐κB, and mitochondrial pathways, ultimately exhausting the CAR‐T cells and reducing their therapeutic efficacy.

Lymphocyte‐specific protein tyrosine kinase (LCK), Zeta‐chain‐associated protein kinase 70 (ZAP‐70), Mothers against decapentaplegic homolog (SMAD), Signal Transducer and Activator of Transcription 3 (STAT3), Casitas B‐lineage Lymphoma (c‐Cbl), Suppressor of Cytokine Signaling (SOCS), Nuclear Factor Kappa‐light‐chain‐enhancer of activated B cells (NF‐κB), Histone Deacetylases (HDACs), Programmed Death‐1 (PD‐1), Cluster of Differentiation 28 (CD28), Interleukin‐2 Receptor (IL‐2R), and Interleukin‐7 Receptor (IL‐7R).

### 2.3 Interaction of TAM and Other Cells in TME

MDSCs and TAMs are both key immunosuppressive populations in TME that limit CAR‐T cell efficacy. While TAMs primarily suppress immunity through cytokine secretion, MDSCs further contribute by causing metabolic dysregulation, producing ROS and arginase‐1, which hinder CAR‐T cell function.^[^ ^75^, ^76^ ^]^ Both populations also express immune checkpoint receptors like PD‐L1, inducing T‐cell exhaustion and impairing CAR‐T cell activity. Although TAMs and MDSCs share roles in immune suppression, their mechanisms differ—TAMs mainly affect cytokine signaling, while MDSCs influence metabolism, such as lactate accumulation, arginine depletion, and altered fatty acid metabolism, and immune checkpoint expression.^[^ ^77^ ^]^ Targeting both populations could improve CAR‐T cell function and therapeutic outcomes in solid tumors.

TAMs and cancer stem cells (CSCs) are known to support each other, contributing to tumor progression and resistance to immunotherapy. CSCs, known for their self‐renewal and resistance to treatments like CAR‐T, interact with TAMs, particularly those with an M2‐like phenotype, to promote tumor growth.^[^ ^78^ ^]^ TAMs secrete cytokines (e.g., IL‐6, TGF‐β) that support CSC self‐renewal and prevent differentiation, thus aiding in tumor progression and metastasis.^[^ ^79^ ^]^ Additionally, TAM‐derived exosomes, which contain proteins, lipids, RNAs (such as miRNAs), and other bioactive molecules, reinforce CSC stemness by transferring these molecules to CSCs, creating a feedback loop that exacerbates CAR‐T therapy resistance.^[^ ^80^ ^]^ Recent studies highlight TAMs may also influence CSC epithelial‐to‐mesenchymal transition (EMT), further complicating treatment.^[^ ^81^ ^]^ This underscores the need for therapies targeting both TAMs and CSCs to improve outcomes in solid tumors.

## 3 Innovative Therapeutic Approaches Targeting TAMs to Enhance CAR‐T Therapy

Among the challenges to CAR‐T cell therapy in solid tumors, TAMs are particularly critical. TAMs contribute to immune suppression and tumor progression, hindering CAR‐T efficacy. Targeting or reprogramming TAMs to enhance their anti‐tumor activity can improve CAR‐T cell function.^[^ ^82^ ^]^ TAMs exhibit significant plasticity, adapting their phenotype based on the local microenvironment. In most solid tumors, they adopt an M2‐like phenotype that promotes tumor growth and suppresses immune responses.^[^ ^83^ ^]^ Thus, targeted deletion of M2‐like TAM or reprogramming TAMs to an M1‐like phenotype with antitumor activity is a promising strategy to enhance treatments like CAR‐T cell therapy.^[^ ^84^ ^]^

### 3.1 Targeted Deletion of M2‐Like TAM

It was reported that CAR‐T cell‐mediated depletion of immunosuppressive M2‐like TAM not only promotes endogenous antitumor immunity but also augments adoptive immunotherapy. A subset of TAMs that express folate receptor β (FRβ) possess an immunosuppressive M2‐like profile. CAR‐T cell‐mediated selective elimination of FRβ+TAMs in the TME results in delayed tumor progression, and prolonged survival in tumor mouse models, highlighting the pro‐tumor role of FRβ+TAMs in the TME and the therapeutic implications of TAM‐depleting agents as preparative adjuncts to conventional immunotherapies that directly target tumor antigens.^[^ ^82^ ^]^

### 3.2 Cytokine Modulation

Modifying the cytokine profiles within the tumor microenvironment is one of the effective strategies used in TAM reprogramming. Some researchers have tried to shift exogenous cytokine IFN‐γ, which causes M1 polarization, or just using vectors to amplify the production of M1 cytokines (GM‐CSF) locally, which have been shown to support the inhibition rather than the progression of tumor growth in TAM phenotypes.^[^ ^85^ ^]^ Cytokine modulation to reprogram TAMs toward an M1 phenotype shows promise, but challenges include controlling local cytokine production and ensuring long‐term stability without exacerbating inflammation or tissue damage.

### 3.3 Targeted Delivery Systems

Sophisticated delivery methods, especially those utilizing nanoparticles, enable direct modification of TAM polarization with little off‐target impact. These nanoparticles can be designed to transport siRNA or shRNA specific to and silencing M2‐associated genes, or they can inhibit pathways selectively active in M2‐like TAMs with small molecule inhibitors promoting an M1‐like phenotype.^[^ ^86^ ^]^ Nanoparticle delivery methods offer targeted TAM polarization with minimal off‐target effects, but challenges in precise targeting within the tumor microenvironment and potential long‐term risks require further investigation.

### 3.4 Metabolic Reprogramming

Recent research highlights that TAMs' reliance on aerobic glycolysis contributes to immune suppression in the TME by producing lactate, which inhibits CAR‐T cell activation. Targeting glycolytic enzymes like HK2 or LDHA could shift TAM metabolism, reducing immunosuppression and restoring CAR‐T cell function.^[^ ^87^ ^]^

Immunometabolic therapies enhance CAR‐T cell efficacy by reprogramming both CAR‐T and TAM metabolism. By promoting oxidative phosphorylation and glucose availability in CAR‐T cells, and disrupting TAM‐induced glycolysis and lactate production, this dual approach can improve CAR‐T cell persistence, activation, and anti‐tumor responses while reducing exhaustion.^[^ ^88^ ^]^ Dual immunometabolic therapies show promise, but balancing CAR‐T persistence with avoiding off‐target effects that could worsen tumor heterogeneity or inflammation remains a challenge.

### 3.5 Epigenetic Reprogramming

Epigenetic modulation could shift TAMs from an M2‐like immunosuppressive phenotype to an M1 anti‐tumor phenotype, enhancing CAR‐T cell function. Targeting histone deacetylases (HDACs) or DNA methyltransferases (DNMTs) may restore the immune balance in the tumor microenvironment, offering a potential strategy to overcome TAM‐mediated immune suppression and improve CAR‐T therapy efficacy.^[^ ^89^, ^90^ ^]^ Targeting HDACs and DNMTs may shift TAMs to an anti‐tumor phenotype, but the long‐term stability and safety of these modifications in solid tumors need further research.

### 3.6 Signaling Intervention

CSF1R inhibition, using agents like pexidartinib, reduces M2‐like TAMs and reprograms the immune landscape to enhance anti‐tumor responses.^[^ ^91^, ^92^ ^]^ Similarly, targeting the CCL2/CCR2 axis to block TAM recruitment shows promise in reducing tumor growth. Additionally,^[^ ^93^, ^94^, ^95^ ^]^ PI3Kγ inhibitors can shift TAMs to a more tumoricidal phenotype, improving anti‐tumor immunity in preclinical models.^[^ ^96^, ^97^ ^]^ However, their clinical translation remains uncertain due to potential off‐target effects and the complex plasticity of TAM phenotypes in the tumor microenvironment.

### 3.7 Novel CAR‐T Designs

Novel CAR‐T therapies targeting both tumor cells and TAMs address challenges in the tumor microenvironment and TAM‐driven immunosuppression. Dual CAR‐T cells, with two distinct CAR molecules, target tumor‐specific antigens and TAMs,^[^ ^98^ ^]^ while Tandem CARs (TanCARs) combine binding domains for tumor antigens (e.g., HER2 or EGFR) and M2‐like macrophage markers (e.g., CD206), enhancing CAR‐T cell activation and TAM elimination, improving efficacy.^[^ ^99^, ^100^ ^]^ Trifunctional CAR‐T cells further target tumor heterogeneity by adding macrophage markers to prevent tumor escape. CAR‐Ts targeting both PD‐L1 on TAMs and tumor antigens block immunosuppressive signals while activating anti‐tumor immunity.^[^ ^101^ ^]^ Additionally, next‐generation CAR‐T cells engineered to secrete cytokines help reprogram the TME, overcoming barriers like hypoxia, ECM, and nutrient limitation.^[^ ^102^ ^]^ Novel CAR‐T therapies targeting both tumor cells and TAMs offer promising strategies to overcome immunosuppressive barriers, but TAM plasticity and off‐tumor toxicity remain significant challenges. While dual and trifunctional CAR‐T designs enhance efficacy, careful consideration of immune dysregulation is crucial for clinical success.

## 4 Emerging Technologies to Enhance CAR‐T Therapy

### 4.1 scRNA‐seq

scRNA‐seq has been pivotal in revealing the transcriptional diversity of TAMs within TME. It uncovers distinct TAM subpopulations, including those expressing immune checkpoint ligands like PD‐L1, which contribute to CAR‐T cell exhaustion. TAMs often adopt an M2‐like phenotype in solid tumors, secreting immunosuppressive cytokines that hinder CAR‐T cell function.^[^ ^103^, ^104^, ^105^ ^]^

In addition to profiling TAMs, scRNA‐seq can assess how CAR‐T cells respond to TAM‐derived signals. This allows researchers to track CAR‐T cell exhaustion and identify strategies to overcome TAM‐induced suppression, such as reprogramming TAMs toward an M1 phenotype to enhance CAR‐T therapy effectiveness.^[^ ^106^ ^]^

### 4.2 Single‐Cell Proteomics

Single‐cell proteomics offers deep insights into CAR‐T cell and TAM interactions in TME. While scRNA‐seq reveals gene expression, single‐cell proteomics analyzes protein markers, enabling a detailed profile of TAMs and CAR‐T cells. Techniques like CyTOF and single‐cell protein arrays help identify key signaling molecules and immune checkpoint receptors on CAR‐T cells, which are regulated by TAMs and contribute to CAR‐T cell exhaustion.^[^ ^107^, ^108^, ^109^ ^]^

Moreover, single‐cell proteomics uncovers the secretion of cytokines, chemokines, and growth factors by both TAMs and CAR‐T cells, influencing CAR‐T cell survival and function. It also identifies metabolic changes, such as TAM‐induced glycolysis, that hinder CAR‐T cell energy production and efficacy in solid tumors.^[^ ^16^ ^]^ These findings open new avenues for targeting immune checkpoints and metabolic pathways to improve CAR‐T therapy outcomes in solid tumors by counteracting TAM‐mediated suppression.

### 4.3 Imaging Techniques

Recent advances in imaging technologies have revolutionized the study of TAM and CAR‐T cell interactions in solid tumors, enabling detailed live‐cell imaging. These techniques allow for clear examination of TAM and CAR‐T cell localization, trafficking, and functional responses in their natural setting.

#### 4.3.1 Multiphoton Microscopy

Multiphoton microscopy is a powerful imaging technique that uses near‐infrared light to visualize CAR‐T cells and TAMs in vivo within TME. It provides high‐resolution, 3D images, enabling real‐time monitoring of the movement, infiltration, and clustering of immune cells. For instance, multiphoton microscopy has facilitated the study of interaction between CAR‐T cells and TAMs within solid tumors, demonstrating the TAM‐mediated inhibitory effects on CAR‐T cell migration and their preferences for localization to immune suppressive regions of the tumors.^[^ ^110^ ^]^

#### 4.3.2 Confocal Microscopy

Confocal microscopy is a valuable tool for studying TAM‐CAR‐T cell interactions at high resolution. It provides detailed optical sections of tumor tissue and, when combined with fluorescent markers, reveals the localization and interactions of CAR‐T cells and TAMs. Confocal imaging can, for instance, be applied in monitoring the expression of immune checkpoint molecules like PD‐L1 on TAMs and their interactions with CAR‐T cells, yielding key insights into the underlying mechanisms of CAR‐T cell exhaustion.^[^ ^101^ ^]^

#### 4.3.3 Positron Emission Tomography (PET) and Magnetic Resonance Imaging (MRI)

In vivo monitoring of CAR‐T cell distribution and effectiveness in solid tumors is achieved through PET and MRI. PET imaging, with radiolabeled tracers targeting CAR‐T cells or TAMs, allows non‐invasive tracking of immune cell populations, such as CAR‐T cell localization with anti‐CD19 antibodies.^[^ ^111^ ^]^ MRI provides detailed anatomical information on the tumor and TME changes.^[^ ^112^ ^]^ Together, these techniques enable longitudinal studies of CAR‐T therapy and its impact on the TME.

## 5 In Vivo Models for Studying TAM‐CAR‐T Cell Interactions

In vivo models are essential for studying TAM‐CAR‐T cell interactions in solid tumors. They replicate the tumor microenvironment, allowing researchers to evaluate the impact of TAMs on CAR‐T cell activity, persistence, and therapy. Various models, each with distinct advantages and limitations, are used to investigate these interactions.

### 5.1 Mouse Tumor Models

Syngeneic mouse models, where tumors from the same species are implanted, are commonly used to study TAM‐CAR‐T cell interactions. These models enable the examination of immune cell behavior in an immunocompetent host, revealing TAMs' role in regulating CAR‐T cell responses.^[^ ^113^ ^]^ Researchers can assess CAR‐T cell effectiveness by tracking tumor progression, CAR‐T cell persistence, and immune checkpoint marker expression.^[^ ^113^ ^]^ Additionally, the impact of TAM depletion, such as using CSF1R inhibitors, on CAR‐T cell activity and tumor response can be evaluated.^[^ ^114^ ^]^

### 5.2 Xenograft Models

In a xenograft model, tumor cells from a human patient are implanted into mice, such as nude mice or NSG mice, which do not have a functional immune system. These models allow the study of human CAR‐T cell therapy in vivo and evaluate the interaction of human TAMs, derived from patient tumors, with CAR‐T cells in a humanized TME.^[^ ^115^ ^]^ Xenograft models enable the study of the impact that TAMs have on CAR‐T cell trafficking, persistence, the ability to kill tumors, as well as the possibility of overcoming TAM‐mediated resistance mechanisms through therapeutic interventions.^[^ ^116^, ^117^ ^]^

### 5.3 Humanized Mouse Models

Humanized mouse models, such as HIS (Human Immune System) and BLT (Bone marrow, Liver, Thymus) mice, are essential for studying human immune responses within TME. HIS mice are generated by transplanting human immune cells, including T cells, B cells, and macrophages, into immunodeficient mice. This is typically achieved by engrafting human peripheral blood mononuclear cells (PBMCs) or hematopoietic stem cells (HSCs), providing a functional human immune system that facilitates the study of cancer therapies like CAR‐T cells.^[^ ^118^ ^]^ BLT mice, which represent a more advanced model, involve the implantation of human fetal tissues (bone marrow, liver, and thymus) into immunodeficient mice, thereby offering a more comprehensive reconstitution of the human immune system.^[^ ^119^ ^]^ Both HIS and BLT models are crucial for understanding human‐specific immune dynamics and the role of TAMs in modulating CAR‐T cell efficacy, thereby overcoming the limitations of traditional murine models.

### 5.4 3D Tumor Organoids

Apart from conventional animal models, 3D tumor organoid models are developing as a useful aid for the investigation of TAM‐CAR‐T cell interactions. Tumor organoids are created from the patient's tumor biopsy, and they can recapitulate the structure and cellular diversity of the original tumors. These models in vitro enable the co‐culture of CAR‐T cells and TAMs in an enhanced physiological context, revealing how TAMs modulate the activity of CAR‐T cells within a controlled system.^[^ ^120^ ^]^ Although 3D organoid models can't completely mimic the intricacies of in vivo tumor microenvironments, they provide a robust framework for examining the molecular processes of TAM‐CAR‐T cell interactions, as well as evaluating numerous prospective therapeutic strategies.^[^ ^121^ ^]^

## 6 Clinical Translation of CAR‐T Cell Therapy in Solid Tumors

While CAR‐T therapy has seen success in blood cancers, its application in solid tumors faces significant challenges due to the complex TME and the need for tailored strategies.

### 6.1 Clinical Safety Considerations

CAR‐T cell therapy in solid tumors faces safety risks like CRS and neurotoxicity. CRS causes systemic inflammation, while neurotoxicity leads to neurological issues. Strategies such as CAR constructs with inducible “safety switches” are being explored to control CAR‐T cell activity and mitigate side effects. A notable advancement is the development of a “suicide gene” system using the inducible caspase 9 (iC9) safety switch. This system allows for the rapid elimination of CAR‐T cells in case of severe adverse events.^[^ ^122^ ^]^ Clinical trials have demonstrated its efficacy in mitigating CRS and neurotoxicity, providing a safeguard for patients undergoing CAR‐T therapy.^[^ ^123^ ^]^

### 6.2 Feasibility of Solid Tumor Treatment

Challenges like dense ECM, hypoxia, and immune suppression by TAMs and MDSCs hinder CAR‐T cell effectiveness. Strategies, including ECM‐modifying CAR‐T cells and those engineered to survive in hypoxic environments, are being tested. Additionally, combining CAR‐T with TAM‐depleting agents has shown promise in cancers like ovarian and colorectal. For instance, a study demonstrated that combining nattokinase, a natural fibrinolytic enzyme, with mesothelin‐targeted CAR‐T cells improved therapeutic efficacy in solid tumors by remodeling the ECM and promoting CAR‐T cell infiltration.^[^ ^124^ ^]^

### 6.3 Landscape of Clinical Trials

CAR‐T cell therapies are currently being evaluated across several solid tumors, including glioblastoma, non–small‐cell lung cancer (NSCLC), and mesothelioma. In glioblastoma, a Phase I trial using intrathecal delivery of bivalent CAR‐T cells targeting EGFR and IL13Rα2 demonstrated good tolerability and reduced tumor enhancement in all patients.^[^ ^125^ ^]^ Another Phase I study administering a single intravenous dose of autologous EGFRvIII‐directed CAR‐T cells to 10 patients with recurrent glioblastoma was also feasible and safe, with one patient maintaining stable disease for over 18 months.^[^ ^126^ ^]^ In NSCLC, a Phase I trial assessing EGFR‐targeted CAR‐T cells in advanced relapsed or refractory cases reported favorable safety and feasibility, with one patient achieving a partial response lasting over 13 months and six patients maintaining stable disease.^[^ ^127^ ^]^ In malignant pleural mesothelioma, intrapleural infusion of mesothelin‐targeted CAR‐T cells showed encouraging persistence and clinical benefit, particularly when combined with PD‐1 blockade, underscoring the therapeutic potential of localized CAR‐T and checkpoint inhibitor combination therapy in solid tumors.^[^ ^128^ ^]^These trials highlight key challenges in CAR‐T therapy for solid tumors, including antigen heterogeneity, tumor evolution, and immune escape. The immunosuppressive tumor microenvironment and metabolic competition also limit CAR‐T cell persistence. Addressing these issues will require multi‐target CAR‐T designs, personalized approaches, and strategies to modify the tumor microenvironment for improved long‐term efficacy.

### 6.4 Regulatory and Manufacturing Challenges

Solid tumor CAR‐T products often necessitate greater personalization and functional optimization, which increases manufacturing complexity and cost. The regulatory pathways for solid tumor indications are still evolving, with agencies such as the FDA and EMA requiring robust evidence of durable benefits and acceptable safety profiles. This necessitates rigorous trial design and the implementation of standardized manufacturing and quality control protocols.

## 7 Conclusion

In conclusion, this review described the issues created by TAMs in solid tumors as they relate to the resistance of CAR‐T cell therapies. Through various means of cytokine secretion, immune checkpoints, and metabolic competition, TAMs maintain a highly immunosuppressive environment that antagonizes CAR‐T cell activity. Additionally, PTMs like TAM‐induced phosphorylation, ubiquitination, acetylation, and glycosylation further deepen CAR‐T cell exhaustion and immune avoidance, decreasing the effectiveness of therapy. Modulating TAMs in the TME, such as cytokine signaling intervention, TAM metabolic reprogramming, and novel CAR‐T designs, can greatly improve the efficacy of CAR‐T therapies in solid tumors. The rapid development of single‐cell omics and in vivo imaging opens new avenues for studying these challenges and refining strategies to address them.

## Conflict of Interest

The authors declare no conflict of interest.

## Author Contributions

X.Z. and H.Z. contributed equally to this work. X.Z. and H.Z. conceived the study, drafted the manuscript, and generated the figures and tables. Y.W.W. and L.C. supervised the study, provided critical revisions, and edited the manuscript. Z.‐C.L., L.‐J.L., X.‐G.C. and X.D. contributed to literature review and manuscript preparation.
